# Statin suppresses the development of excessive intimal proliferation in a Kawasaki disease mouse model

**DOI:** 10.14814/phy2.70096

**Published:** 2024-10-18

**Authors:** Yusuke Motoji, Ryuji Fukazawa, Ryosuke Matsui, Makoto Watanabe, Yoshiaki Hashimoto, Noriko Nagi‐Miura, Tadashi Kitamura, Kagami Miyaji

**Affiliations:** ^1^ Department of Cardiovascular Surgery Kitasato University School of Medicine Tokyo Japan; ^2^ Department of Pediatrics Nippon Medical School Tokyo Japan; ^3^ Laboratory for Immunopharmacology of Microbial Products Tokyo University of Pharmacy and Life Sciences Tokyo Japan

**Keywords:** *Candida albicans* water‐soluble substance, coronary artery lesions, Kawasaki disease, statin, vascular remodeling, vasculitis

## Abstract

Kawasaki disease (KD) causes vascular injury and lifelong remodeling. Excessive intimal proliferation has been observed, resulting in coronary artery lesions (CALs). However, the mechanisms underlying vascular remodeling in CAL and statin treatment have not been comprehensively elucidated. This study aimed to investigate the effects of statins on vascular remodeling using a KD mouse model. *Candida albicans* water‐soluble substance (CAWS) was intraperitoneally injected in 5‐week‐old male apolipoprotein‐E‐deficient mice. They were categorized as follows (*n* = 4): control, CAWS, CAWS+statin, and late‐statin groups. The mice were euthanized at 6 or 10 weeks after injection. Statins (atorvastatin) were initiated after CAWS injection, except for the late‐statin group, for which statins were internally administered 6 weeks after injection. Elastica van Gieson staining and immunostaining were performed for evaluation. Statins substantially suppressed the marked neointimal hyperplasia induced by CAWS. Additionally, CAWS induced TGFβ receptor II and MAC‐2 expression around the coronary arteries, which was suppressed by the statins. KD‐like vasculitis might promote the formation of aneurysm by destroying elastic laminae and inducing vascular stenosis by neointimal proliferation. The anti‐inflammatory effects of statins might inhibit neointimal proliferation. Therefore, statin therapy might be effective in adult patients with KD with CAL by inhibiting vascular remodeling.

## Graphical abstract

Kawasaki disease‐like vasculitis might promote the formation of aneurysm by destroying elastic laminae and inducing vascular stenosis by neointimal proliferation. The anti‐inflammatory effects of statins might inhibit neointimal proliferation owing to the suppression of TGFβ expression. Statin therapy might be effective in adult Kawasaki disease patients with CAL by inhibiting vascular remodeling.

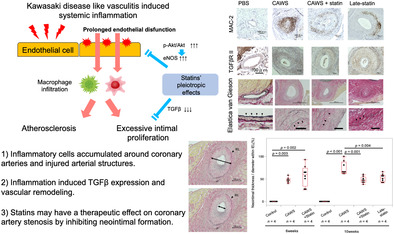

## 1 INTRODUCTION

Kawasaki disease (KD), a systemic acute inflammatory disease, develops in infants and induces coronary artery lesions (CALs), such as coronary artery stenosis and aneurysms (Fukazawa et al., 2020; Kawasaki et al., 1974). CAL is estimated to occur in approximately 25% of patients with KD without appropriate treatment (Newburger et al., 2016); however, the incidence of cardiovascular sequelae has declined from 16.7% in 1983 to 2.3% in 2017 owing to treatment advancements (Fukazawa et al., 2020; Seki & Minami, 2022). However, more than 50 years after the discovery of KD, an estimated 10,000 to 20,000 patients have reached adulthood with residual CAL in Japan (Ae et al., 2020; Fukazawa et al., 2020). While acute‐phase KD treatment has advanced considerably with the use of immunoglobulin therapy and steroids, chronic‐phase treatment still lacks an established evidence‐based method. Therefore, the urgent requirement to establish appropriate treatment methods is evident (Fukazawa et al., 2020). CAL is a pathological lesion, which includes the disruption of the normal vascular structure in the acute phase of vasculitis, and aneurysms develop because of blood pressure‐induced vascular ballooning. Approximately half of the coronary aneurysms regress within 1 year; however, several residual disease cases have been reported (Kato et al., 1996). Excessive intimal malformation and migration and transformation of tunica media vascular smooth muscle cells into the intima due to elastic plate structure disruption are the histologic hallmarks of CAL lesions (Amano et al., 1979; Ishikawa et al., 2022). Neointimal proliferation in aneurysmal coronary arteries lead to negative remodeling in the subacute to chronic phase, resulting in vascular lumen narrowing (Ogawa et al., 2004; Orenstein et al., 2012). To induce vascular remodeling, transformed vascular smooth muscle cells produce growth factors such as transforming growth factor (TGF) β, vascular endothelial growth factor, and platelet‐derived growth factor (PDGF) (Chang et al., 2021; Chen et al., 2022; Gao et al., 2018; Suzuki et al., 2000). KD vasculitis increases the risk of developing atherosclerosis during early adulthood, leading to vascular remodeling and coronary artery stenosis in CAL lesions (Fukazawa et al., 2007; Suzuki et al., 2000). Statins, the first‐line drugs for atherosclerosis, exhibit pleiotropic effects (Oesterle et al., 2017) and are expected to suppress vascular remodeling through anti‐inflammatory effects (Fukazawa et al., 2020; Isselbacher et al., 2022). Hamaoka et al. (2010) reported the efficacy of statin therapy on continuous post‐inflammatory vascular remodeling late after KD in clinical studies. Statins demonstrate potential as a key treatment for CAL patients. The American Heart Association Kawasaki Disease Statement and Japanese guidelines state that statins are considered preemptive in CAL patients (recommendation class IIb, level of evidence C) (BW et al., 2017; Fukazawa et al., 2020). Clinical trials are being conducted in Japan and the USA to evaluate the efficacy of atorvastatin in patients with acute KD with coronary artery aneurysm. Studies suggest that statins can be well tolerated and effective, and Phase III trials are strongly recommended in the future. In experimental studies, a mouse model of Kawasaki‐like vasculitis using *Candida albicans* water‐soluble substance (CAWS) has been widely used (Hirata et al., 2012; Nagi‐Miura et al., 2006; Ohno, 2003). CAWS deposits in the adventitia of the aortic root and causes a large and persistent inflammatory cell infiltrate around the coronary arteries (Hamaoka‐Okamoto et al., 2014; Miyabe et al., 2019). Our group reported, for the first time, that CAWS vasculitis in mice with apolipoprotein‐E‐deficiency (Apo E−/−) causes chronic macrophage cell infiltration of the aortic root and aortic tissue and that statins may exert an anti‐inflammatory effect by suppressing inflammatory cell infiltration (Motoji et al., 2019). Furthermore, we reported that statins might contribute to the homeostasis of vascular endothelial cells, which are crucial in preventing macrophage cell infiltration (Motoji et al., 2022). In this study, we aimed to examine how CAWS vasculitis affects vascular remodeling in the coronary arteries and whether statins have suppressive effects on coronary artery stenosis.

## 2 MATERIALS AND METHODS

All mouse experimental procedures were performed in accordance with the Guidelines for Animal Experiments of the Nippon Medical School (Tokyo, Japan). This animal experiment was approved by the Animal Experimental Ethics Committee of Nippon Medical School (No: 2020–029). All experiments were performed in accordance with the guidelines from Directive 2010/63/EU of the European Parliament on the protection of animals used for scientific purposes, the NIH guidelines (guide for the care and use of laboratory animals), and ARRIVE guidelines (NC3Rs Reporting Guidelines Working Group, 2010; Percie du Sert et al., 2020).

### 2.1 Animals

Five‐week‐old male C.KOR/StmSlc‐ApoEshl mice were purchased from Sankyo Labo Service Co., Ltd. (Tokyo, Japan). Mice were maintained in a 12‐h light/12‐h dark cycle at 20–24°C with 40%–70% humidity. All mice were maintained under specific pathogen‐free conditions according to the guidelines for animal care of the National Institute of Infectious Diseases in Tokyo (Science Council of Japan, n.d.). We used only male mice to prevent any effects of sex. Water and food were provided ad libitum. Mice were fed a commercial rodent diet (MF; Oriental Yeast Co., Ltd., Tokyo, Japan) until 7 weeks of age, after which they were transitioned to a high‐fat diet containing 0.15% cholesterol (test diet 58Y1 Lard 60% kcal Diet; PMI Nutrition International, Tokyo, Japan). Mouse feeding and drug administration were performed by Y. Motoji and R. Fukazawa. The data in this study are derived from the same group of animals as in one of our previously published studies (Motoji et al., 2019) but are entirely independent of that study's analyses and objectives.

### 2.2 CAWS and statin preparation

CAWS was prepared from the *C. albicans* strain NBRC1385, as previously described (Uchiyama et al., 1999). Briefly, 5 L of C‐limiting medium was maintained in a glass incubator for 2 days at 27°C and 400 rpm, with air being supplied at a 5 L/min rate. An equal volume of ethanol was subsequently added, and the mixture was allowed to stand overnight, after which the precipitate was collected and dissolved in 250 mL of distilled water. Ethanol was added, and the mixture was left to stand overnight once again. The precipitate was subsequently collected and dried with acetone to obtain CAWS.

The HMG‐CoA inhibitor atorvastatin calcium hydrate (atorvastatin) was provided by Sankyo Ltd. (Tokyo, Japan). Atorvastatin was crushed and dissolved in 0.5% (w/v) methylcellulose 400 solution (Waco Fujifilm Corporation, Tokyo, Japan) and sterilized. Statins were orally administered simultaneously to the high‐fat diet (from 7 weeks of age) at a 10‐mg/kg/day dose, which was determined based on the clinical dose administered to humans. Statins were directly administered using an oral sonde to eliminate errors in statin doses administered due to differences in individual food consumption.

### 2.3 Experimental procedures

**FIGURE 1:**
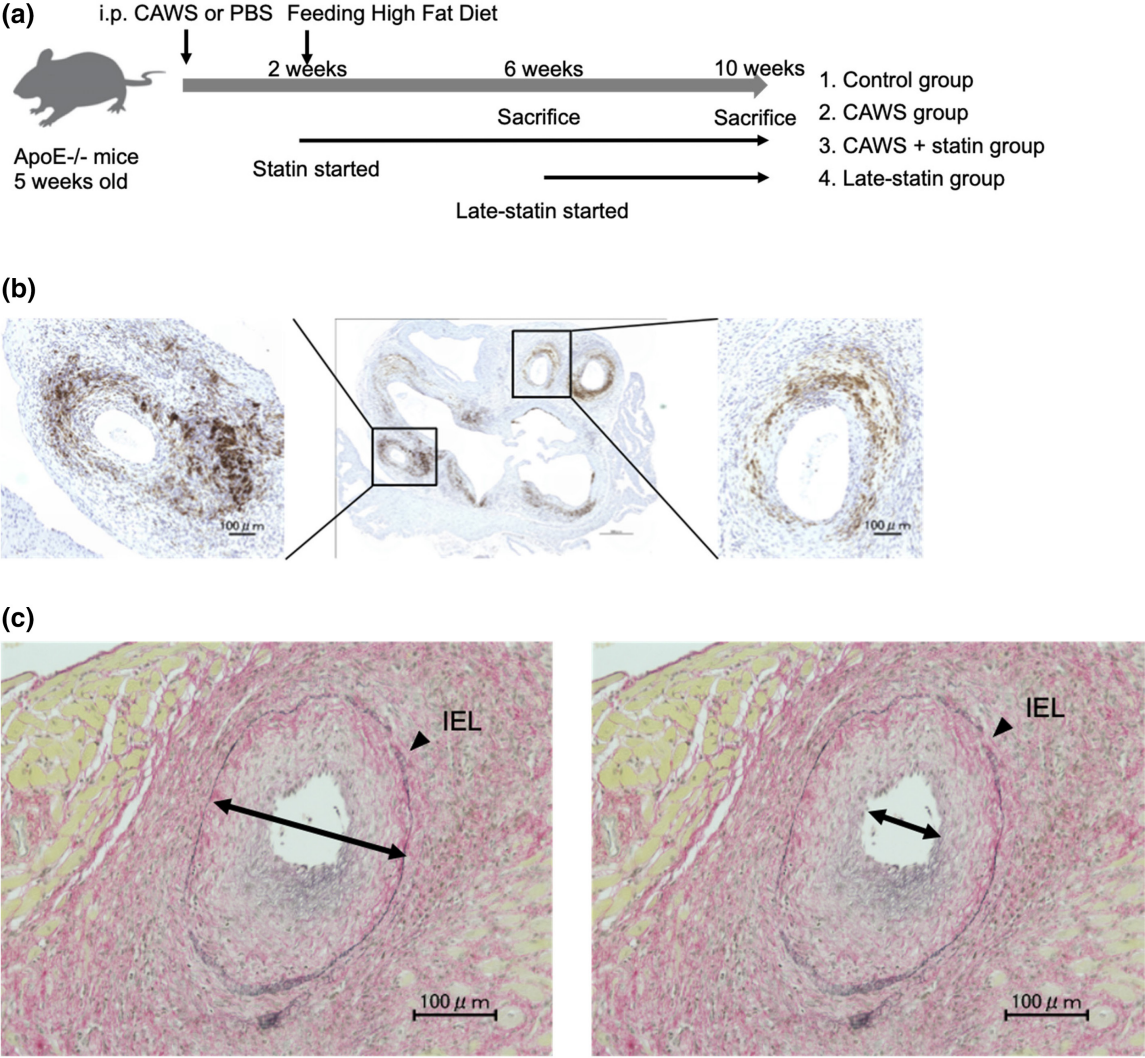
(a) Experimental procedure. *Candida albicans* water‐soluble substance (CAWS; 4 mg for five consecutive days; the control group received phosphate‐buffered saline (PBS) instead) was intraperitoneally injected into 5‐week‐old Apo E−/− mice to induce Kawasaki disease‐like vasculitis (the control group received PBS instead). The diet of all mice was changed to a high‐fat diet to promote atherosclerosis after 2 weeks of CAWS administration. Mice were categorized into four groups: 1. control group, 2. CAWS group, 3. CAWS + statin group and 4. late‐statin group. Statins (10 mg/kg/day) were dissolved in 0.5 mL of 0.5% methylcellulose solution and subsequently orally administered with a sonde. The control group was administered a methylcellulose solution without statin. The CAWS and CAWS + statin groups daily received statins from 2 weeks after CAWS administration to the end of the experiment. The late‐statin group daily received statins from 6 weeks after CAWS administration to the end of the experiment. Mice were euthanized after 6 and 10 weeks of CAWS administration, and the samples were collected. (b) Histological findings at the coronary arteries. Histological findings at the aortic root after CAWS administration for 10 weeks were recorded. In one base specimen, two coronary arteries were identified, enlarged, and analyzed. Immunostaining for macrophage cells and Elastica van Gieson (EVG) staining for elastic fibers around the coronary arteries were performed. Anti‐MAC‐2 antibody staining. Right and left; scale bar, 100 μm. Middle; scale bar, 500 μm. (c) Definition of the percentage of stenosis at the distance between the internal elastic lamina (IEL). Coronary artery neointimal thickness was selected as an indicator of intima formation and defined as the thickened intima extending into the IEL. To reduce the bias owing to the presence or absence of aneurysms, the coronary percentage of stenosis between IELs was comparatively evaluated. Scale bar, 100 μm.

Mice were divided into four groups for comparison (Figure 1a). Mice were euthanized at 6 (11 weeks of age) and 10 (15 weeks of age) weeks after CAWS injection. The life cycle of mice is assumed to be considerably shorter than that of humans. At 8 weeks of age, male mice reach sexual maturity. Fifteen weeks of age in mice corresponds to adulthood in human years (Flurkey et al., 2007; Hirata et al., 2012; Palliyaguru et al., 2021). Because statins are not administered to infants in clinical practice, we designed a model where statins are administered in early adulthood. All mice were fed a high‐fat diet containing 0.15% cholesterol for 2 weeks. Five mice from each group were euthanized 10 weeks after CAWS injection (at 15 weeks of age), and their hearts and aorta were retrieved for analysis. To euthanize mice, an overdose of pentobarbital was administered intraperitoneally (150 mg/kg).

### 2.4 Division of mice into groups

1. Control group: 5‐week‐old Apo E−/− mice were intraperitoneally injected with phosphate‐buffered saline instead of CAWS. Instead of statins, the same volume of methylcellulose solution was orally administered to mice daily in the same manner.
2. CAWS group: CAWS (4 mg/mouse) was intraperitoneally injected into 5‐week‐old Apo E−/− mice for five consecutive days, as performed by Hashimoto et al. (2019). The mice were orally injected with a methylcellulose solution daily instead of statin in the same manner.
3. CAWS + statin group: mice were orally administered statin daily from 2 weeks after CAWS administration (from 7 weeks of age) until the last day of the experiment.
4. Late‐statin group: mice were started on statin orally from 6 (11 weeks of age) weeks after CAWS administration until the last day of the experiment. We designed a model where statins are administered from early adulthood, given that statins have no indication in infancy in clinical practice.

### 2.5 Assessing the area of aortic root horizontal transection

Mice were anesthetized, and the heart and aortic root were embedded in paraffin and identified in serial sections (5 μm). Subsequently, they were stained using Elastica van Gieson (EVG) staining/immunostaining as previously described (Motoji et al., 2019). The following analyses were performed using the Hybrid Cell Counting System (KEYENCE) and the KEYENCE BZX Analyzer (Osaka, Japan). Trained observers (39, 21, 14, 13, and 11 years of experience) blinded to treatment performed image analysis. The specimen was transected through the level of the Valsalva sinus, and three aortic valves were identified. In that slice, the coronary arteries were identified. The percentage of coronary artery lumen stenosis was measured in two coronary arteries per animal and the statistical analyses was based on the number of animals/group as the independent biological replicates. Intimal thickness was selected as an indicator of intimal formation, which was defined as the thickened intima afferently overhanging the inside of the internal elastic lamina (IEL). When assessing neointima thickness, the presence of an aneurysmal target coronary artery may considerably impact the results. Hence, the coronary lumen stenosis percentage between the IEL was comparatively evaluated. Intima length at four sites along the coronary artery was measured and averaged. This was followed by evaluation of two coronary arteries for each animal and presentation of the average values of the stenosis rate of each coronary artery. The percentage of stenosis at the distance between the IEL was calculated using the formula [(thickness between IELs ‐ thickness of neointima)/thickness between IELs x 100 (%)] as a quantitative parameter for coronary artery stenosis, based on the evaluation used by Suganuma et al. (2021) (Figure 1c). The length of the images was determined using the ImageJ software version 1.54 (National Institute of Health, Bethesda, MD, USA).

### 2.6 Assessing CAWS‐induced elastin degradation

Elastin breaks were scored on the following scale: score 0, no interruption of elastic fibers; score 1, elastic breaks ≤ 10; 2, elastic breaks >10; and score 3, obscuration or disappearance of elastic fibers. Interruptions in elastin fibers and reappearance of fibers were considered elastin breaks and expressed as the total number of breaks and panvasculitis as previously described (Suganuma et al., 2021). Three locations were scored per coronary image. The scores were totaled for each animal, and the effects of vasculitis were scored.

### 2.7 Evaluating macrophage cell and TGFβR II expression

The area (mm^2^) stained via immunostaining was measured, and the area ratio to the peri‐coronary artery tissue area was calculated. The peri‐coronary area was defined as the area covering 1.2 times the coronary artery diameter. The lesion area was quantified and analyzed using the Hybrid Cell Counting System (KEYENCE) and KEYENCE BZX Analyzer (Osaka, Japan). A trained observer blinded to treatment performed the image analysis (Centa et al., 2019). The aortic root sections were immunohistochemically analyzed to detect macrophage cell and TGFβ receptor II (TGFβR II) expression. The macrophage cells and their fractions, which represent inflammatory cells, were identified using immunostaining. Additionally, immunostaining for TGFβR II was performed because TGFβ signaling is reportedly involved in vascular remodeling in KD vasculitis (Lee et al., 2015).

Accordingly, the following sheep anti‐rabbit antibodies were used: anti‐Galectin 3 (MAC‐2) antibody (1/250, 60 min at room temperature, Abcam; ab76245 RRID: AB_2265782, UK), and anti‐TGFβR II antibody (1/500, 60 min at room temperature, Abcam; ab186838 RRID: AB_2728775, UK), markers specific for macrophages. The sections were further treated with secondary antibodies and developed using horseradish peroxidase‐conjugated DAB substrate (Abcam; ab236446).

### 2.8 Statistical analysis

Statistical data were expressed as median (upper and lower quartiles) or mean ± standard deviation (SD). Statistical analyses were performed using JMP (version 17, SAS Institute Inc., Cary, NC, USA). The Kruskal–Wallis test was used to analyze statistical differences among groups. The Wilcoxon test was used as a post‐hoc test to compare values between both groups when significance was detected. Statistical significance was set at a *p*‐value <0.05. Spearman's rank correlation was used for all correlation analyses, and Spearman's coefficients are denoted by *ρ*.

## 3 RESULTS

### 3.1 Macrophage cell immunostaining using anti‐galectin 3 (MAC‐2) antibody in the peri‐coronary arteries

**FIGURE 2:**
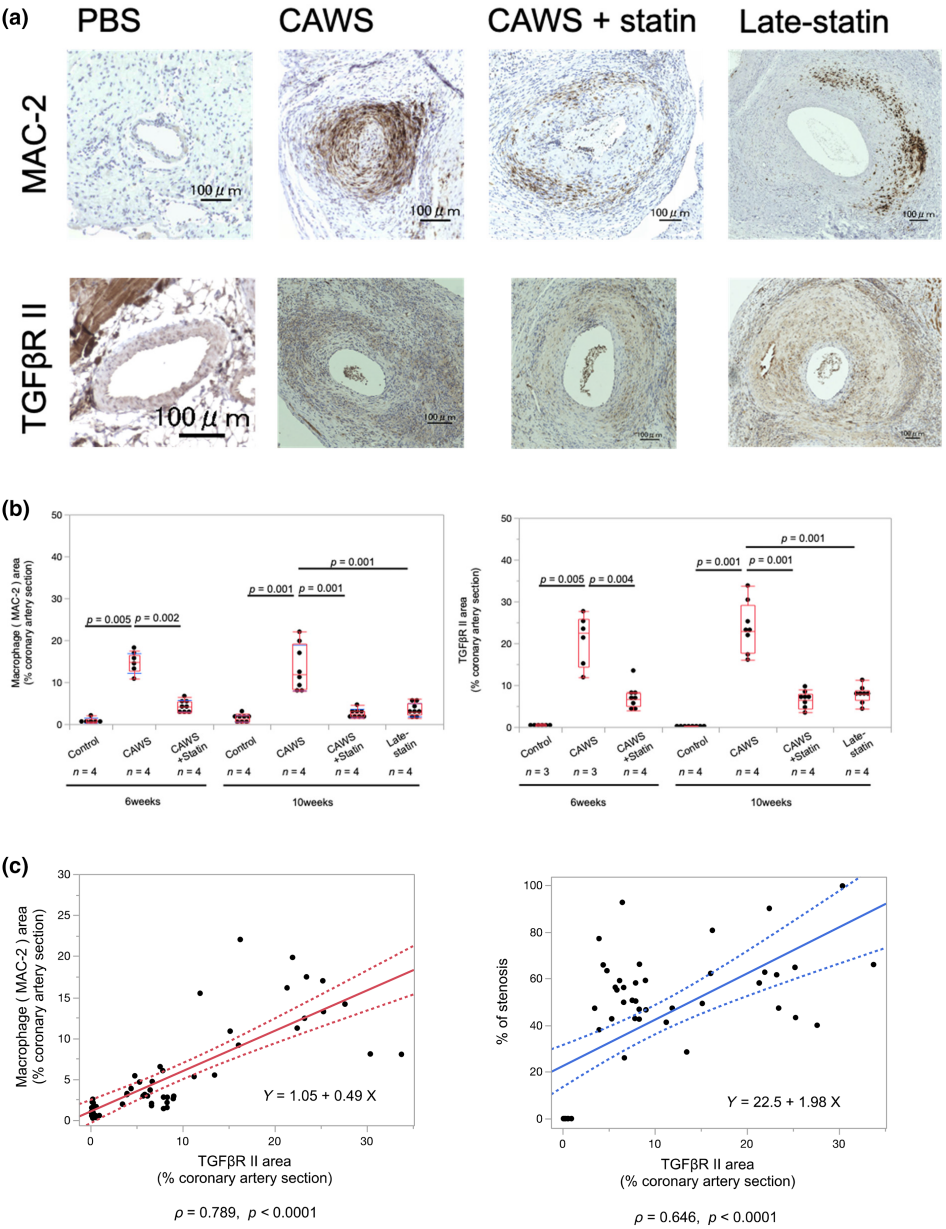
(a) Immunostaining findings for coronary arteries. The area ratio of MAC‐2 and transforming growth factor (TGF) βR II expression around coronary arteries was calculated. *Candida albicans* water‐soluble substance (CAWS) enhanced MAC‐2 and TGFβR II expression. Statin treatment suppressed macrophage cell infiltration and vascular remodeling at the coronary arteries; these effects of statins were also observed in the late‐statin group. Scale bar, 100 μm. (b) Macrophage cell invasion area ratio and TGFβ receptor II expression area ratio. Macrophage cell infiltration area and TGFβR II expression area ratios were calculated and compared between the groups. Area ratios were expressed as percentages. CAWS substantially promoted macrophage cell infiltration and TGFβR II expression at 6 and 10 weeks after CAWS administration. Additionally, statins considerably inhibited macrophage cell infiltration and TGFβR II expression 6 and 10 weeks after CAWS administration. The inhibitory effect of statins was also observed in the late‐statin group. Data were analyzed by the Kruskal–Wallis test. The Wilcoxon test was corrected with a post‐hoc test. The values are mean ± SD. *n* = 3–4 mice. All data were generated from one experiment in the same timeframe. (c) Correlations between macrophage cell, TGFβR II, and neointimal percentage. Correlation coefficient scatter diagrams are shown. The neointimal percentage at the distance between the internal elastic lamina was markedly associated with the macrophage cell invasion area ratio and TGFβR II expression area ratio in coronary arteries. Scatter plots showing Spearman correlations and correlation coefficients (*ρ*) between TGFβR II, macrophage cell invasion area ratio, and the neointimal percentage. The same sites were observed and analyzed for two coronary arteries observed in one individual specimen. *n* = 3–4 mice. All data were generated from one experiment in the same timeframe.

Horizontal sectioned specimens at the aortic root were immunostained, and the coronary arteries observed at the root were enlarged and analyzed at two regions per specimen (Figure 1b). At 6 and 10 weeks after CAWS administration, a remarkable accumulation of macrophage cells was observed around the coronary arteries in the CAWS group compared to that in the control group (6 weeks: 0.9 ± 0.6% vs. 14.5 ± 2.3% [*p* = 0.005]; 10 weeks: 1.4 ± 0.8% vs. 13.5 ± 5.5% [*p* = 0.001]; Figure 2a,b).

Macrophage cell accumulation was significantly suppressed in the CAWS + statin group compared to that in the CAWS group (6 weeks: 14.5 ± 2.3% vs. 4.3 ± 1.4% [*p* = 0.002]; 10 weeks: 13.5 ± 5.5% vs. 2.6 ± 1.0% [*p* = 0.001]; Figure 2a,b).

The macrophage cell infiltration area ratio in the late‐statin group was significantly lower than that in the CWAS group (*p* = 0.001) but was not significantly different from that in the CAWS + statin group (*p* = 0.372).

### 3.2 TGFβR II antibody immunostaining in the peri‐coronary arteries

The TGFβR II expression area ratio was significantly increased in the CAWS group compared to that in the control group 6 and 10 weeks after CAWS administration (6 weeks: 0.4 ± 0.3% vs. 20.8 ± 6.1% [*p* = 0.005]; 10 weeks: 0.2 ± 0.2% vs. 23.7 ± 6.2% [*p* = 0.001]; Figure 2a,b).

The CAWS + statin group exhibited a significant decrease compared to the CAWS group 6 and 10 weeks after CAWS administration (6 weeks: 20.8 ± 6.1% vs. 7.1 ± 2.9% [*p* = 0.004]; 10 weeks: 23.7 ± 6.2% vs. 6.2 ± 1.9% [*p* = 0.001]; T Figure 2a,b).

The TGFβR II expression ratio of the late‐statin group was 7.8 ± 2.0%, which was significantly lower than that of the CAWS group (*p* = 0.001) but not significantly different from that of the CAWS + statin group (*p* = 0.207).

### 3.3 Correlations between the macrophage cell, TGFβR II, and neointimal percentages

Correlations were examined between the macrophage cell invasion area ratio, TGFβR II expression area ratio, and neointimal percentage at the distance between the IEL. A significant correlation was observed between the macrophage cell invasion area ratio and TGFβR II expression area ratio in the coronary arteries (*ρ* = 0.789; *p* < 0.0001) and between the neointimal percentage at the distance between the IEL and TGFβR II expression area ratio (*ρ* = 0.646; *p* < 0.0001; Figure 2c).

### 3.4 Effect of statin on the CAWS‐induced elastin degradation

**FIGURE 3:**
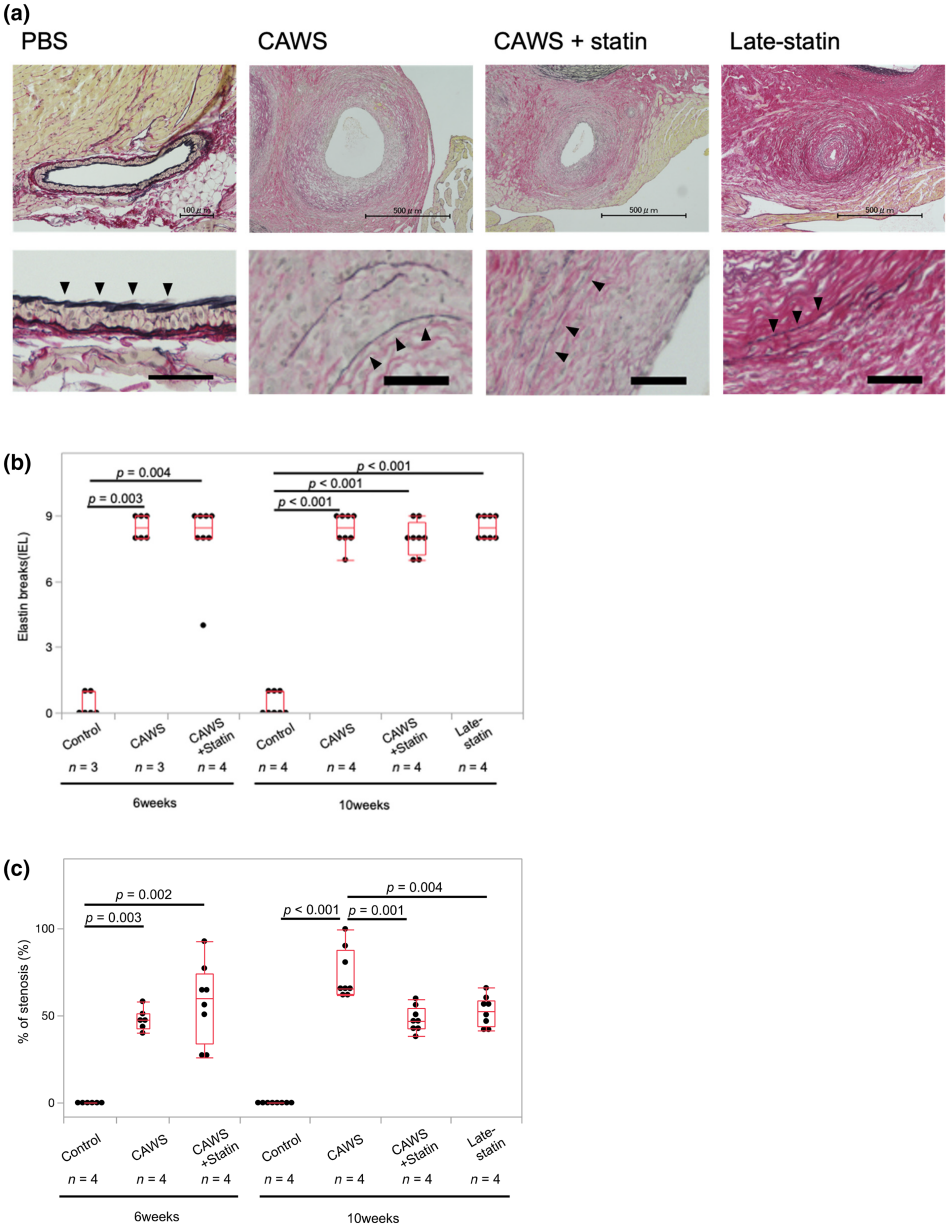
(a) Structural evaluation of the elastic fibers in the coronary arteries using Elastica van Gieson staining. These images show the histological findings after 10 weeks of *Candida albicans* water‐soluble substance (CAWS) administration. CAWS vasculitis disrupted the elastic fiber structure of the coronary arteries. No recovery was observed using statin medication once the elastic fiber structure was broken. Top: Elastica van Gieson (EVG) staining of the coronary artery; transverse section. Scale bar, 100–500 μm. Bottom: Close‐up view of the elastic fiber structure. Black arrow: Internal elastic lamina. Scale bar, 50 μm. (b) Elastin breaks in the elastic lumina. Elastin breaks in the elastic lumina were semi‐quantitatively evaluated to evaluate the effect of statins on CAWS‐induced elastin breaks; EVG staining of coronary arteries from mice treated with phosphate‐buffered saline (PBS), CAWS and CAWS + statin. CAWS‐induced vasculitis damaged the elastic fiber structure of the coronary arteries. Statin therapy did not improve them. Data were analyzed by the Kruskal–Wallis test. The Wilcoxon test was corrected with a post‐hoc test. The values are mean ± SD. *n* = 3–4 mice. All data were obtained from a single experiment in the same time frame. (c) Percentage of stenosis at the distance between the internal elastic lamina (IEL). The intimal formation was defined as the thickened intima afferently overhanging the inside of the IEL. As an indicator of intimal formation, we chose to use intimal thickness. Neointima in the coronary arteries was markedly increased by CAWS vasculitis. Statins did not suppress neointimal proliferation 6 weeks after CAWS administration. However, statin therapy considerably suppressed neointimal proliferation at 10 weeks. Notably, neointimal proliferation was also suppressed in the late‐statin group. Data were analyzed by the Kruskal–Wallis test. The Wilcoxon test was corrected with a post‐hoc test. The values are mean ± SD. *n* = 4 mice. All data were generated from one experiment in the same timeframe.

The CAWS group exhibited significantly higher scores for breaking and weakening of the elastic fibers than the control group (6 weeks: 0.3 ± 0.5% vs. 8.5 ± 0.5% [*p* = 0.003]; 10 weeks: 0.4 ± 0.5% vs. 8.4 ± 0.7% [*p* < 0.001]; Figure 3a,b). Elastin rupture scores in the CAWS + statin and late‐statin groups were not significantly different compared to those in the CAWS group (*p* > 0.1; Figure 3a,b). The elastic fiber structure was not repaired by statin once disrupted by CAWS vasculitis.

### 3.5 Statins suppress excessive intimal proliferation

When assessing neointimal thickness, the presence of an aneurysmal target coronary artery may significantly affect the results.

Therefore, the ratio of the neointimal diameter between IELs was comparatively evaluated. The CAWS group exhibited significantly enhanced neointimal growth in the coronary arteries compared to the control group (6 weeks: 0.0 ± 0.0% vs. 47.5 ± 6.2% [*p* = 0.003]; 10 weeks: 0.0 ± 0.0% vs. 73.4 ± 14.8% [*p* < 0.001]; Figure 3a,c). After 6 weeks of CAWS administration, no significant suppression of coronary neointimal proliferation by statins was observed (*p* = 0.220); however, a significant suppression was observed 10 weeks after CAWS administration (10 weeks: 73.4 ± 14.8% vs. 47.8 ± 7.1% [*p* < 0.001]; Figure 3a,c). Notably, neointimal proliferation was significantly suppressed in the late‐statin group (52.4 ± 8.6%) compared to that in the CAWS group (*p* = 0.004).

## 4 DISCUSSION

The current study presents three major findings: (1) CAWS vasculitis was histologically confirmed to destroy the arterial three‐layer structure by accumulating inflammatory cells around coronary arteries; (2) it also induced stenosis by promoting neointimal proliferation in the lumen of the impaired coronary arteries; and (3) statins might exert a therapeutic effect on coronary artery stenosis by suppressing neointima formation.

Previous studies have demonstrated that CAWS vasculitis caused chronic inflammatory cell infiltration, predominantly of macrophage cells, at the aortic root in an ApoE−/− atherosclerosis mouse model and that statins markedly reduced inflammatory cell infiltration in animal experiments (Motoji et al., 2019). CAWS vasculitis affects vascular endothelial cells, impairing endothelial nitric oxide synthase (eNOS) production, which is crucial for vascular homeostasis. This condition is associated with macrophage cell infiltration into the tissue and chronic macrophage cell persistence (Motoji et al., 2022). In the inflammatory microenvironment that KD induces, vascular remodeling (tissue destruction and reconstruction) is triggered by several pathways from the macrophage (Chang et al., 2021; Goumans & Ten Dijke, 2018). Endothelial‐to‐mesenchymal transition (EndMT), the differentiation process of vascular endothelial cells that make up the vascular lumen into non‐epithelial mesenchymal cells, is crucial in the vascular remodeling process (Dejana & Lampugnani, 2018; Yamashiro et al., 2023). TGFβ secreted by the macrophage cells activates EndMT (Cooley et al., 2014; Ma et al., 2020; Yamashiro et al., 2023). Additionally, PDGF /PDGFRβ, which is involved in cross‐talk with TGFβ, regulates cell migration in EndMT (He et al., 2022; Kandasamy et al., 2020).

Hyperactivated platelets isolated from patients with KD promote differentiation into vascular smooth muscle cells via the PDGF /PDGFRβ axis (Yahata et al., 2014; Zhang et al., 2020). In this KD‐like vasculitis mouse model, PDGFRβ overexpression was also observed in the thickened medial layer and vascular endothelium (An et al., 2023). These findings suggest that macrophage cells and TGFβ are closely associated with neointimal hyperplasia in coronary arteries in CAWS‐induced vasculitis models.

In this study, CAWS vasculitis considerably increased the area ratio of TGFβR II expression around the coronary arteries and was notably suppressed by statin treatment. Moreover, the expression area ratio of TGFβR II was markedly correlated with the macrophage cell area ratio and neointima/vessel diameter ratio of coronary arteries. Statins did not restore the elastic fiber structure destroyed by vasculitis but significantly inhibited tissue infiltration of macrophage cells in the mouse model of CAWS vasculitis. Although imaging assessment is required for the acute phase and over time, in the present study, we found that macrophage cells infiltrated coronary arteries from the adventitia toward the intima. It was suggested that statins reduce macrophage infiltration in the intima, which may be closely related to improving vascular endothelial cell function through statin‐enhanced expression of eNOS (Motoji et al., 2022). Statins substantially suppressed TGFβ expression and might have a therapeutic effect on vascular remodeling, particularly in the progression of stenotic lesions.

We examined whether statin initiation in patients with KD and CAL has a therapeutic effect, such as preventing aneurysm development. Notably, neointimal proliferation was nearly suppressed in the late‐statin group as in the CAWS + statin group. These findings suggest that initiating statin treatment after the onset of CAL formation in patients with KD may still yield a sufficient therapeutic effect.

In the CAWS + statin group, statins were designed to be continuously administered from infancy; however, statins have not been approved for administration in infants (Miura et al., 2021). In the present study, statin administration was initiated in individuals in the late‐statin group at 11 weeks of age, which corresponds to approximately 20 years of age in humans (Dutta & Sengupta, 2016). The fact that neointimal proliferation was similarly suppressed in this group may suggest a reasonable recommendation for oral statin therapy in adult patients with KD and CALs.

The present study has certain limitations. CAWS vasculitis is highly similar to KD vasculitis but not an identical experimental model. However, clinical studies have noted several similarities between the two models. As previously discussed, using CAWS vasculitis in this study is meaningful because similar mechanisms operate in CAWS and KD vasculitis. ApoE −/− mice are prone to easier development of atherosclerosis and increased susceptibility to vascular endothelial cell dysfunction, raising concerns (Miura et al., 2021). The use of the ApoE −/− model was expected to enhance the observed therapeutic effects of statins. This statin dose does not markedly reduce serum low‐density lipoprotein (LDL) levels in mice (Motoji et al., 2019; Wang et al., 2014; Zhang et al., 2017). The experiment did not demonstrate the LDL‐lowering effect of statins as anticipated (data not shown). In the future, confirming the pleiotropic effects of statins in more general experimental systems such as DBA/2 mice is necessary. In the present study, all mice were of male sex. In Japan, the incidence of KD is slightly higher in males than in females; however, the reason for this is unclear (Fukazawa et al., 2020). To fairly evaluate statin efficacy, future experiments on female individuals are necessary. Finally, this study does not clarify the interaction between statins and the TGFβ pathway. Therefore, further studies are necessary to elucidate the mechanism.

In summary, a possible KD model, CAWS vasculitis, was related to CAL formation via induction of macrophage cell tissue infiltration around the coronary arteries and chronic inflammation. Statins demonstrated the potential to prevent coronary restenosis by inhibiting neointimal proliferation in CALs. The CAL frequency was increased in immunoglobulin‐refractory patients. This study is expected to lead to the recognition of the efficacy of statins as a treatment modality to prevent coronary restenosis in early adulthood.

## AUTHOR CONTRIBUTIONS

Conceptualization: Y.M. and R.F.; methodology and validation: Y.M., R.F., R.M., M.W., N.N.‐M, and Y.H.; formal analysis: Y.M., R.M., M.W., Y.H, and R.F.; investigation: Y.M.; resources: Y.M, R.F., N.N.‐M, and K.M; data curation: Y.M. and R.F.; writing—original draft preparation: Y.M.; writing—review and editing: R.F. and T.K.; visualization: Y.M.; supervision: Y.M., T.K., and K.M.; project and administration: Y.M. and R.F.; funding acquisition: Y.M. and R.F. All authors have read and agreed to the published version of the manuscript.

## FUNDING INFORMATION

This work was supported by Grant‐in‐Aid for Scientific Research Japan (JP12K34567), Grant‐in‐Aid for Early‐Career Scientists (24K18864), and a research grant 2023 for young medical doctors and healthcare professionals from SRL, Inc.

## CONFLICT OF INTEREST STATEMENT

The authors declare no conflicts of interest.

## ETHICS STATEMENT

The Animal Care and Use Committee of Nippon Medical School approved the animal study protocol (approval number 2022‐029).

## Data Availability

The datasets generated and/or analyzed during the current study are available from the corresponding author upon reasonable request.
